# Sperm Selection Procedures for Optimizing the Outcome of ICSI in Patients with NOA

**DOI:** 10.3390/jcm10122687

**Published:** 2021-06-18

**Authors:** Kaan Aydos, Oya Sena Aydos

**Affiliations:** 1 Department of Urology, Reproductive Health Research Center, School of Medicine, University of Ankara, 06230 Ankara, Turkey; 2 Department of Medical Biology, School of Medicine, University of Ankara, 06230 Ankara, Turkey; saydos@gmail.com

**Keywords:** testicular azoospermia, non-obstructive azoospermia, sperm selection, sperm, cryopreservation, in vitro maturation

## Abstract

Retrieving spermatozoa from the testicles has been a great hope for patients with non-obstructive azoospermia (NOA), but relevant methods have not yet been developed to the level necessary to provide resolutions for all cases of NOA. Although performing testicular sperm extraction under microscopic magnification has increased sperm retrieval rates, in vitro selection and processing of quality sperm plays an essential role in the success of in vitro fertilization. Moreover, sperm cryopreservation is widely used in assisted reproductive technologies, whether for therapeutic purposes or for future fertility preservation. In recent years, there have been new developments using advanced technologies to freeze and preserve even very small numbers of sperm for which conventional techniques are inadequate. The present review provides an up-to-date summary of current strategies for maximizing sperm recovery from surgically obtained testicular samples and, as an extension, optimization of in vitro sperm processing techniques in the management of NOA.

## 1. Introduction

To date, although testicular spermatozoa from patients with non-obstructive azoospermia (NOA) have been used widely for intracytoplasmic sperm injection (ICSI), this method’s effectiveness still has potential for further improvement. NOA is characterized by the absence of any spermatozoa, whether dead or alive, in the ejaculate due to reduced or nonexistent sperm production in the testicle [1]. Testicular fine needle aspiration (FNA or testicular sperm aspiration—TESA) is an effective and non-invasive method used to obtain sperm, especially from patients with obstructive azoospermia [2]. Although its simpler and less traumatic features have made FNA the preferred method, testicular sperm extraction (TESE) is the treatment of choice for patients with NOA, with a satisfactory number of successful spermatozoa retrieved in approximately half of the patients. In the conventional TESE procedure, the testis is exposed through a small incision in the tunica albuginea, and multiple biopsies are taken randomly [3]. However, microTESE carried out at high magnification under an operating microscope allows visualization of whitish, larger, and more opaque seminiferous tubules likely to contain mature germ cells [4]. Although not randomized, most studies have reported that the sperm recovery rate from microTESE is superior to that from conventional TESE [5,6,7]. In fact, a recent controlled, randomized study verified the efficacy of microTESE compared with that of TESE in retrieving spermatozoa from patients with NOA [8]. In addition to the surgical technique, however, in vitro extraction of sperm from surgically excised testicular tissue or tubules is also important for obtaining spermatozoa of sufficient quality and quantity for use in ICSI.

## 2. Processing and Selection of Surgically Retrieved Sperm for ICSI

### 2.1. Mechanical Processing of Testicular Tissue

The goal of TESE treatment in patients with NOA is to retrieve spermatozoa suitable for ICSI from the testicular tissue obtained by surgical intervention. Different methods have been described for processing TESE specimens in the laboratory. The most preferred tissue-processing procedure is mechanical treatment of testicular tissue pieces by shredding and mincing with fine needles, scissors, or glass slides [9,10,11]. In addition to the shredding, tubule pieces cut into short lengths can be squeezed into the medium with the help of a bent pipette [12,13]. The suspension can then be processed using either the swim-up or density gradient centrifugation method. In the former, following the sedimentation of tissue fragments, the most motile spermatozoa swim up the medium; in the density gradient method, during centrifugation, sperm cells are separated according to swimming rate while moving through discrete layers of density gradients [14,15]. Both methods have their advantages. Verheyen et al. compared rough shredding, fine mincing, vortexing, and crushing methods to evaluate the efficiency of obtaining a maximum number of sperm from testicular biopsy specimens, and fine mincing of testicular tissue followed by discontinuous Percoll centrifugation was found to be the most effective method of isolating a pure fraction of spermatozoa available for ICSI as well as cryopreservation [16]. However, the risk of cell loss through the discrete layers in Percoll gradient separation cannot be ignored. Therefore, if too few cells are present, it may be preferable to carefully mince the testicular tissue with fine forceps or microscissors and centrifuge the entire suspension immediately [10,17]. As another option, Haimov-Kochman et al. suggested that leaving tissue fragments from TESE in medium for 10 min and then centrifuging the supernatant could yield rapid sperm recovery without wasting time shredding the tissue [18]. Interestingly, when the supernatant was spermatozoa-negative, no spermatozoa were present in the tissue either. Therefore, this method makes it possible to quickly predict the success of TESE. Nevertheless, when it comes to choosing the optimal method, in addition to the structural features of the testicular tissue, personal experience and laboratory facilities should also be taken into consideration.

### 2.2. Use of Erythrocyte-Lysing Buffer (ELB)

One of the most frequently encountered problems during the search for spermatozoa in fragmented TESE specimens is abundant erythrocyte infiltration. Attempting to identify rare spermatozoa among dense erythrocyte clusters is time consuming and associated with a reduced recovery rate. The use of erythrocyte lysing buffer (ELB) for the elimination of red blood cells present in the mechanically shredded testicular tissues of patients with NOA was first described by Verheyen et al. [19]. Resuspending the testicular sperm pellet in ELB (155 mM NH4Cl, 10 mM KHCO3, and 2 mM EDTA; pH 7.2) yielded recovery of additional motile spermatozoa in a shorter time. Treatment of TESE samples with ELB shortens the processing time and increases the success of cell retrieval without decreasing the fertilization potential of the embryo [17]. However, treatment of sperm suspensions with ELB has also been shown to impair sperm quality [20]. When spermatozoa were incubated in ELB for 10 min, their motility and viability decreased and DNA fragmentation increased. This effect of ELB on sperm may be due to the fact that the same mechanism of disruption in ammonium equilibrium responsible for the lysis in erythrocytes is also present in the sperm. In addition, osmotic stress caused by the chemical structure of this buffer may damage the plasma membrane and alter sperm metabolism [21]. Conversely, Soygur et al. showed that ELB itself and the cellular stress caused by erythrocyte lysis did not have detrimental effects on the survival of ejaculated sperm [22]. According to their protocol, however, ELB incubation was only allowed for 5–10 s, so there may not have been enough time for damage to occur. Despite the fact that the potential influence of this buffer on sperm parameters remains uncertain, at present, ELB medium is widely used during testicular germ cell extraction to clean the cellular suspension from erythrocytes that interfere with visualization [23,24].

### 2.3. Enzymatic Digestion

In TESE, the goal of mechanically shredding tissues is to free the germ cells that are trapped in seminiferous tubules or adhered to the tissue. However, digestion of tissues using enzymes can be expected to facilitate the release of gametes by loosening the cellular contacts in the tubular wall. In fact, collagenase has been shown to provide sufficient dissolution in the tissue without decreasing cell viability [25] (Table 1). This was also confirmed by Salzbrunn et al. for cryopreserved testicular tissue, and enzymatic preparation of tissues using collagenase type AI provided high yields of vital spermatids and spermatozoa [26]. Subsequently, Fischer et al. reported the first pregnancy using spermatozoa extracted using the same method in ICSI [27]. However, when compared to type AI, collagenase type IV has been shown to be more effective in isolation and recovery of viable spermatozoa and round spermatids from testicular samples [28]. Type IV collagenase is preferred for the processing of testicular tissue, since type IV collagen is specifically localized in the basement membrane of the seminiferous tubules and within the extracellular matrix (ECM) layers [29]. On the other hand, collagenase type IV is produced by Sertoli cells (SCs), and its target (collagen IV) has been shown to affect the dynamics of SC-tight junctions, which mediates translocation of preleptotene and leptotene spermatocytes residing at the basal compartment of the seminiferous epithelium into the adluminal compartment for further development [30]. However, the exact mechanisms regulating the events of spermiation and sperm release have not yet been clearly elucidated. A disintegrin and metalloproteinase with thrombospondin motifs (ADAMTSs), which belong to the M12 metallopeptidase family, are responsible for the migration of differentiated germ cells through the seminiferous tubule wall by organizing the degradation and reformation of the ECM [31]. In fact, we recently demonstrated that ADAMTS1 and ADAMTS5 protein levels expressed in Sertoli cells were decreased approximately 2-fold in cases of NOA [32]. The effect of enzymatic digestion on the success of sperm retrieval in cases where these proteases are defective is a subject of further research. For these reasons, the use of type IV collagen has been widely preferred to increase sperm recovery rates, because of both its disrupting effect on the integrity of the tubule basement membrane and its ability to break the connections of germ cells with Sertoli cells [33]. During the process, clotting caused by free DNA released from dead cells can be prevented by the addition of DNAase enzyme [28].

Since enzymatic digestion of testicular tissue allows obtainment of an isolated germ cell suspension free of tissue artifacts, its efficiency is higher compared to rough mechanical separation, which causes contamination with large amounts of damaged cells, free nuclei, and residual tissue fragments in the cell suspension. Indeed, spermatozoa retrieval rates (SRRs) between 7% and 33% were reported following enzymatic processing of tissue suspension in TESE cases in which no spermatozoa could be obtained by mechanical mincing [33,34,35,36]. The differences between the SRRs reported in the studies may be due to the experience of the embryologists, the histopathological status of the testicular tissues, and variations in the number of cases. In particular, the time spent and effort expended during the mechanical shredding of seminiferous tubules have significant effects on the chance of sperm detection in the laboratory. The crucial role of laboratory handling, especially in the management of compromised testicular specimens, has been discussed previously [37]. Similarly, Modarresi et al. reported serum follicle-stimulating hormone (FSH) and luteinizing hormone levels as factors that may affect SRRs after enzymatic digestion treatment [33]. On the other hand, collagenase treatment has been shown to increase the cytokine population in the testis by stimulating an immune response, but its effects on spermatozoa remain unclear [38]. In general, processing with enzymatic digestion from testes in which very few spermatozoa are predicted to be present should be considered an effective practice.

### 2.4. Motility Enhancers

The success of ICSI in patients with NOA is closely related to the recovery of motile spermatozoa during TESE. Indeed, fertilization success decreases significantly when immotile spermatozoa are injected into oocytes [39,40]. However, in most patients with NOA, only a small number of spermatozoa with very poor motility or complete immotility can be obtained from minced testicular tissue. Less than 3% sperm motility was reported following the initial processing of biopsy samples [41]. However, the primary goal in the treatment of NOA is to extract spermatozoa with sufficient motility from tissue fragments during TESE for use in ICSI. It has been suggested that viable sperm ratios increase after in vitro culture of testicular cell suspension with various motility enhancers [15] (Table 2). Among the many tested compounds for stimulating sperm motility in vitro, phosphodiesterase (PDE) inhibitors have been the most promising. The most common chemical component used as a motility enhancer in the laboratory is pentoxifylline. Pentoxifylline is a PDE inhibitor of the methylxanthine group shown to induce sperm motility [42]. In human sperm, adenylyl cyclase catalyzes the formation of cyclic adenosine monophosphate (cAMP) from adenosine triphosphate. The role of cAMP in the regulation of sperm motility has been defined [43]. Previous studies have suggested that cAMP is the primary signal for the onset of progressive motility under proper conditions [44]. Compounds such as pentoxifylline, caffeine, and theophylline are known to inhibit cyclic nucleotide phosphodiesterase, which breaks down cAMP to 5′-AMP [45,46]. However, it is not certain that theophylline and caffeine directly stimulate sperm motility by increasing the cAMP compound; they may also act on other enzymes in addition to PDE [43]. Caffeine was shown to increase cytochrome oxidase activity, an important compound of the electron transport chain in mitochondria, by stimulating the cAMP and protein kinase A pathway [47]. Experimental studies revealed that the addition of caffeine to semen samples increased sperm motility and stimulated capacitation and spontaneous acrosome reaction [48,49]. It was also shown that incubation of post-freezing human spermatozoa with caffeine for 15 min increased progressive motility through mitochondrial energy metabolism [50]. However, teratogenic consequences of high-dose caffeine and its derivatives have been established in animal models [51]. It has been suggested that by washing away the motility enhancers, the putative toxic effects on embryo development will disappear; however, it has also been shown that the removal of these compounds by centrifugation may lead to total motility loss [52]. Although the results in current literature are encouraging, the toxic effects of caffeine on embryo development and the potential prevention of these effects require further investigation.

In a comparative study, Mangoli et al. demonstrated that using pentoxifylline in the selection of viable spermatozoa from a testicular non-motile spermatozoa population significantly increased the success of fertilization (62% vs. 41%) and pregnancy (32% vs. 16%) more than the hypo-osmotic swelling test [53]. Pentoxifylline was found to increase the pregnancy success rate of IVF with its stimulating effect on acrosome reaction [54]. Similarly, Kovacic et al. reported that if immotile sperm in TESE tissue were cultured with pentoxifylline for 20 min, 97% of them started to move; hence, vital spermatozoa could be distinguished more easily, the procedure was shortened, and the fertilization rates and numbers of embryos increased [55]. The use of pentoxifylline to stimulate sperm motility in fresh or frozen/thawed suspensions has also been reported in other studies [56,57,58]. Since the cAMP level in spermatozoa with normal motility is sufficiently high to activate the subsequent cascades, PDE inhibitors have the most pronounced effect on sperm with poor motility [59]. This explains why pentoxifylline is rather successful on poorly motile sperms recovered with TESE. Above all, artificial oocyte activation was indicated as the main determining factor for ICSI success, regardless of the restoration of testicular sperm motility after pentoxifylline treatment [58]. Therefore, when evaluating the clinical results of in vitro sperm processing methods, the female factor should also be taken into consideration in certain aspects.

Theophylline, with its similar molecular structure to pentoxifylline, has also been shown to increase sperm motility and fertilizing capacity in in vitro studies [52,60,61]. Ebner et al. reported that when frozen/thawed testicular sperm samples from TESE were treated with theophylline, sperm motility improved in most cases, and the clinical pregnancy rate increased from 23% to 53% [45]. The positive effect of theophylline on sperm motility has also been demonstrated in other studies [62,63]. Although there is concern that motility-enhancing chemicals may have toxic effects on embryo development [64], no evidence of anomalies in offspring has been shown for either theophylline or pentoxifylline [63,65].

Spermatozoa contain various forms of PDEs with different regulatory interactions. Results from in vitro studies with PDE5, which represents a small functional fraction of spermatozoa, are contradictory. Glenn et al. reported that Sildenafil citrate, a widely used PDE5 inhibitor, produced a sustained improvement in sperm motility, but this was accompanied by a significant increase in acrosome-reacted sperm count [66]. Others have reported PDE5 inhibitors to yield similar improvements in motility; however, these studies did not confirm that sildenafil and tadalafil (another PDE5 inhibitor) triggered the acrosome reaction [67,68]. Overall, concentration and exposure time of PDE5 inhibitors have been indicated as the main decisive factors for sperm motility [66,69]. Nevertheless, the early acrosome reaction before reaching the oocyte renders sperm unable to fertilize; this constitutes the main concern. Furthermore, paucity of PDE in spermatozoa, as demonstrated in proteomic studies, may also have led to inconsistent results in in vitro studies [70]. In addition, the scarcity of studies on aberrant PDE expression and the role of biochemical pathways with different molecular interactions prevent further conclusions [71].

### 2.5. Short-Term Culture vs. Simultaneous ICSI

The impact of enhancers on sperm motility in TESE samples may also be related to in vitro incubation conditions. In fact, when the effects of culture time and ambient temperature on sperm motility in testicular tissue samples were evaluated, 24 h incubation at room temperature was found to be ideal for optimal sperm motion [72] (Table 3). Balaban et al. showed that incubating testicular tissue samples in recombinant FSH-supplemented medium for 24 h increased the motility of the spermatozoa and, thus, the success of ICSI [73]. Similarly, Wu et al. evaluated the laboratory and clinical results of fresh and post-freezing culturing of tissue samples taken by TESE from a group of patients with NOA [41]. According to their results, after 24 h of in vitro culture at 37 °C, the number of motile sperm increased remarkably, and sperm reached a maximum motility rate at 72 h. Similar studies reported that sperm motility peaked upon extending the culture period to 48 h [74]. Likewise, post-freezing cultures of TESE samples showed results similar to those of fresh cultures. In contrast, the outcome of in vitro cultures of testicular spermatozoa from patients with NOA has not been confirmed by others to be predictable [11]. However, the main goal in the treatment of NOA is to obtain vital spermatozoa with sufficient motility during a TESE attempt. In this context, Karacan et al. who compared the outcome of 337 ICSI cycles using testicular sperm freshly obtained on the day of or the day before oocyte retrieval or after a freeze/thaw cycle, demonstrated that in the presence of motile spermatozoa, neither the timing of TESE nor the use of post-freezing sperm affected ICSI results [75]. On the other hand, since culturing of testicular sperm for up to 24 h was found to increase DNA fragmentation, it is recommended that retrieved sperms are used without delay [76]. Unfortunately, we do not have enough data to make a reliable comment on whether TESE samples should be taken one day in advance and kept in the laboratory or used fresh simultaneously with oocyte pick-up (OPU).

### 2.6. Motile Sperm Identification with HOST

During the examination of testicular tissue under a microscope, the most important factor determining the vitality of the selected spermatozoa is movement of the tail. In recent years, researchers have used various techniques in attempts to develop an optimal selection method that can directly discriminate living spermatozoa, regardless of their motility, while visualized under a micromanipulator. If all spermatozoa are immotile, hypo-osmotic swelling test (HOST) is an option for choosing a viable one for ICSI. HOST, first described by Jeyendran et al., is a vitality test used to assess the functional integrity of the spermatozoa membrane [77]. Principally, viable but immotile spermatozoa incubated in hypo-osmotic solution are expected to have swollen tails due to osmotic challenge, as their membrane functions are healthy. In a semen sample, the percentage of viable spermatozoa selected with HOST is defined as the HOST score [78]. A randomized and controlled study showed that fertilization and pregnancy rates associated with ICSI increased significantly when viable sperm were selected from among immotile testicular spermatozoa using HOST [79]. Furthermore, the use of testicular spermatozoa with total absence of motility selected with HOST demonstrated pregnancy rates comparable to ejaculate [80]. Others also reported similar results [81,82,83]. Likewise, in cases where immotile sperm cannot be activated with known motility enhancers, HOST is a reliable and effective option for choosing viable spermatozoa for ICSI [84]. On the other hand, low HOST values of spermatozoa, an indicator of impaired membrane integrity, may be associated with increased DNA damage [85]. Therefore, HOST can be a valuable tool for the selection of viable and also DNA-intact spermatozoa [86].

In many couples with low HOST scores, pregnancy rates remain low, either in natural cycles or after conventional IVF [87]. However, even if the HOST score is low, acceptable implantation and pregnancy rates can be achieved with ICSI [88]. In these cases, decreased fecundation rates were explained as the toxic effect of a number of spermatozoa with a low HOST score attached to the zona pellucida [78]. Fertilization can be achieved by bypassing this effect, using a single sperm with ICSI. This proves the importance of choosing a viable spermatozoon using HOST for pregnancy success, regardless of the total HOST score of a whole sperm population. Besides, high occurrences of spontaneously developed tail swellings were reported to affect the accuracy of HOST in determining the viability of frozen-thawed spermatozoa, a drawback of HOST for processed sperm [89]. In addition, HOST is not recommended as a viability test. Since pregnancy could be achieved in the following ICSI trials while HOST demonstrated impaired membrane function, it is recommended to verify the HOST results with vitality tests [90]. Moreover, although HOST is practical, the procedure is time consuming and requires chemicals used in the process to be removed [84]. Loss of viability due to prolonged incubation, further dilution of an extremely small number of testicular sperm in HOST solution, and various methodological modification proposals are other troubles related with the test [91,92]. However, the ability to demonstrate in real time that immotile spermatozoa are not dead still makes HOST an indispensable part of ICSI practice.

### 2.7. Sperm Tail Flexibility Test to Check Sperm Viability

Another method of assessing the viability of immotile spermatozoa is the sperm tail flexibility test. Essentially, this test involves observing whether the sperm tail is moving by mechanical agitation with lateral touch of the microinjection pipette [93]. If the tail moves independently of the head, the immotile sperm is considered alive and can therefore be selected for ICSI. In contrast, a spermatozoon’s tail remaining rigid in response to the same force indicates its non-viability. When sperm were selected based on this method, whether from frozen or fresh testicular tissue samples, the pregnancy and take-home baby percentages using immotile and motile sperm were found to be similar [94]. Mechanical assessment of the viability of immotile spermatozoa is a simple and cost-effective method that avoids the risk of chemical solutions and does not disrupt the structural integrity of the sperm [84]. The main disadvantages of this mechanical touching technique are the scarcity of data comparing its results with those of other techniques and, in particular, its dependence on the personal experience and skill of the practitioner.

### 2.8. Intracytoplasmic Morphologically Selected Sperm Injection (IMSI)

IMSI allows selection of spermatozoa with normal nuclear morphology under ultra-high magnification for use in ICSI [95]. Initially, IMSI was confirmed to significantly increase pregnancy rates, especially in cases with recurrent implantation failure following ICSI [96]. Considering that it increases the implantation and pregnancy rates by 50% and 60%, respectively, the use of IMSI in male factor infertility was encouraged [97]. Later, the effectiveness of IMSI was further emphasized in cases of severe compound sperm disorders [98]. However, a recent meta-analysis found low-quality evidence that IMSI increases the clinical chance of pregnancy; the analysis reported the probability of live birth to be 24% by regular ICSI and between 21% and 33% following IMSI coupled with ICSI [99]. Pregnancy with testicular sperm selected by IMSI has also been reported [100]. In cases with high sperm DNA fragmentation, however, spermatozoa from the testicles doubled the live birth rates compared to ejaculated spermatozoa selected with IMSI (49.8% vs. 28.7%) [101]. The relatively high pregnancy percentages in TESE/TESA cases can be explained by the avoidance of testicular sperm from oxidative DNA damage during epididymal transit. Nevertheless, before making a firm conclusion about the role of IMSI in male factor infertility, further studies are needed to confirm whether the magnified morphological structure of a spermatozoon is an indicator of its functionality.

### 2.9. Laser-Assisted Sperm Selection

Laser-assisted (LA) sperm selection has been suggested as a novel technique to check the viability of immotile spermatozoa. To assess viability with a laser, a direct shot is made to the tip of the sperm tail for approximately 2 ms, using 200 µJ of energy. Curling of the tail following the laser shot indicates that the sperm is viable and can therefore be selected for ICSI [102]. In 2004, for the first time, Aktan et al. reported the selection of viable testicular sperm using a laser system [103]. The viability percentages of the selected immotile spermatozoa were similar to those of HOST. However, compared with randomly selected spermatozoa, the authors reported that fertilization and take-home baby rates of laser-selected spermatozoa increased from 20% to 45% and 5% to 19%, respectively. Subsequently, pregnancy with viable but immotile sperm selected using the LA technique in ICSI was reported [104]. A healthy birth was also achieved with laser-selected immotile but living frozen/thawed spermatozoa [105]. Furthermore, live births were reported with LA selection of pentoxifylline-resistant immotile sperm in cases of Kartagener’s Syndrome [106]. Thus, viable sperm selection using LA technology, which is simpler and faster than HOST and does not require the use of chemical agents, now allows immotile sperm to be cryostored in order to preserve fertility. Apart from the instrument cost and the need for experienced personnel, LA sperm selection is considered a promising method for the future [84].

### 2.10. Birefringence-Based Sperm Selection

Another new strategy proposed to distinguish healthy and viable sperm for use in ICSI is the birefringence-based selection technique. Birefringence is defined as the splitting of a light wave into two unequally reflected waves by an optically anisotropic medium. The orderly longitudinal orientation of the nucleoprotein filaments in the protoplasmic texture of the nucleus and acrosomal complex gives mature sperm a characteristic intrinsic birefringent appearance. By evaluating the birefringence of sperm heads with the use of polarized light microscopy, mature and viable sperm can be selected. When compared with spermatozoa selected with HOST, significantly higher pregnancy rates were found in TESE-ICSI cases in which birefringent-headed spermatozoa were used (11% vs. 45%) [107]. Similarly, Gianoroli et al. reported that implantation and pregnancy rates from ICSI cycles were significantly higher when testicular sperm selection was performed using the birefringent method compared to couples using conventionally selected sperm [108]. Although available results from birefringent-selected sperm appear to indicate a better option than conventional methods or HOST, currently, there are not enough existing studies to validate this [109]. Cost and equipment supply in clinical practice are other issues that require resolution.

### 2.11. Microfluidics-Assisted Sperm Sorting

Besides the aforementioned methods, one alternative to functional spermatozoa separation techniques from semen samples has been the use of microfluidics [110]. For this purpose, special devices have been designed in which seminal fluid and media flow simultaneously through a microscopic channel without a physical barrier between them [111]. Due to the unique characteristics of microfluidics, the motile sperm in the sample swim into the parallel-flowing media. A comparative study showed that microfluidic sorting of semen allows highly motile sperm selection with minimal DNA fragmentation compared to standard processing methods [112]. Unfortunately, few studies have examined the use of microfluidic systems for sperm selection in testicular cell suspensions. Recently, a new microfluidic system has been developed to facilitate rapid and efficient sperm isolation from TESE samples [113]. This system processes testicular tissue extract in two successive modules. The first module is a spiral microchannel that separates sperm from red blood cells and cellular debris through microfluidics using inertial forces. In the second module, excess media is removed by means of a hollow fiber membrane designed for mammalian cell isolation, thus enriching the suspension. This system yielded an 8-fold increase in sperm identification time due to the low output volume of the cell suspension and the almost complete elimination of non-sperm bio-particles. However, it remains to be explained whether there is sperm loss in microfluidic processing, especially in samples containing very rare spermatozoa. Loss of immotile but viable sperm can also be a disadvantage of this procedure. The promising initial results have yet to be confirmed by further studies in terms of clinical practice.

### 2.12. Raman Spectroscopy-Assisted Sperm Retrieval

Raman spectroscopy (RS) has been used as a feasible and reliable method for the identification of seminiferous tubules with spermatogenesis in testicular tissues from humans and rats [114,115,116]. RS is a laser-scattering technique that provides information about the internal structure of molecules through chemical fingerprints [117]. Although the available data indicate that RS may be useful in real-time intra-operative distinguishing of seminiferous tubules containing full spermatogenesis during microTESE, a special probe designed for this purpose has not yet been developed. In addition, further studies are needed to investigate the extent of laser-induced testicular tissue damage, confirm genetic safety, and optimize the technical parameters of this system. However, whether microscopy-assisted or combined with microfluidics, RS may be a promising diagnostic tool capable of detecting the molecular characteristics of sperm [118,119].

### 2.13. Fluorescence-Activated Cell Sorting (FACS)

FACS is based on the isolation of living spermatozoa labeled with fluorophore-conjugated antibodies from seminal fluid when irritated by a laser [120]. In a pilot study, testicular spermatozoa could be isolated using this technique in cases of NOA where sperm recovery could not be achieved in previous TESE attempts [121]. However, alterations in cell viability due to fluorophores and antibodies, the cost of the system, high cell loss, and its time-consuming nature are potential limiting factors for the use of FACS in testicular cell selection for ICSI [122].

Comparison of different methods for viable sperm recovery by TESE is shown in Table 4.

### 2.14. Other Emerging Technologies for Predicting Spermatogenesis in the Testes

In addition to the testicular sperm extraction methods mentioned above, various other techniques have been attempted; they are expected to identify spermatogenesis foci precisely and rapidly but are not yet used in routine clinical practice. Multiphoton microscopy (MPM), based on low-energy infrared laser technology, is a technique that uses radiated energy as intracellular autofluorescence. A study in rats using an MPM laser showed that seminiferous tubules with or without sperm could be distinguished in real time depending on the differences in fluorescence [123]. The same group later found an 86% concordance between human testicular biopsy results and MPM diagnoses [124]. In another study, ex vivo testicular tissues from rats were imaged with full-field optical coherence tomography, which uses white light interference microscopy; thus, spermatogenesis within the seminiferous tubules could be identified without the use of contrast or a fixative [125]. Although these novel technologies represent a promising tool for predicting outcomes in cases of NOA, sperm functionality results have yet to be verified.

Although other techniques (e.g., annexin V magnetic-activated cell sorting, hyaluronic acid binding, Zeta method, and mechanisms based in sperm guidance) have also been used in the selection of ejaculated spermatozoa, their efficiency and safety for testicular sperm have not yet been investigated [126].

## 3. Cryopreservation of Surgically Retrieved Sperm for ICSI

Sperm can be retrieved with TESE in approximately 35–52% of men with NOA [127]; however, in cases where pregnancy cannot be achieved, repeated TESE in subsequent cycles does not ensure sperm retrieval in every case of NOA with the same success. Under such circumstances, sperm cryopreservation has made an important contribution to the medical and social rehabilitation of couples by protecting their fertility. Establishing that the length of the storage time in liquid nitrogen does not affect the quality of spermatozoa (or, consequently, the fertilization potential) has allowed the widespread practice of sperm cryopreservation [128].

### 3.1. Fresh vs. Frozen/Thawed TESE

Even though ICSI is traditionally performed using fresh sperm, ready-to-use spermatozoa may not always be available when the oocyte is picked up from the partner, especially if it requires harvesting from the testis. In this context, performing TESE one day before oocyte retrieval or using cryopreserved testicular sperm provides several advantages. First, since sperm can be frozen at any time, there is no need for synchronized OPU planning. Thus, scheduling ICSI cycles and freezing testicular sperm eliminates the risks of ovulation induction and the unnecessary cost of canceled cycles [75]. When TESE is not repeated, the extra expense of a new surgical intervention is also avoided. Moreover, in repeated ICSI cycles, the use of frozen residual spermatozoa from fresh TESE cycles prevents re-surgical trauma of the testicle. Cryopreservation of tissue samples taken with TESE also saves time for further treatment of women who are not eligible for implantation that day. For example, changes in endometrial receptivity have been demonstrated as a contributing factor in recurrent implantation failures [129]. Postponing the embryo transfer may give the woman an opportunity to prepare for the best time for implantation. Likewise, injury to the endometrium may also increase the success of implantation in subsequent cycles [130,131]. Other benefits of sperm cryopreservation are the availability of spermatozoa when ovarian stimulation begins and the opportunity to perform a programmed embryo biopsy to eliminate those with anomalies from ICSI [56], ensuring sperm storage before vasectomy, and making use of sperm banking and sperm donation [132,133]. However, simultaneous TESE on the day of OPU has the advantages of preventing sperm loss after thawing and avoiding sperm damage or loss of quality due to cryopreservation, as explained later [134]. Additionally, if sperm is retrieved successfully, the need for a second course of treatment is eliminated, as OPU and subsequent ICSI will be completed on the same day.

Nevertheless, in many studies, the use of cryopreserved testicular spermatozoa in ICSI has yielded results comparable to or better than those of fresh cycles [135,136,137]. When compared to those with fresh spermatozoa, the cumulative miscarriage rate was significantly lower [4% vs. 14.7%) and the cumulative live birth rate was significantly higher (34.7% vs. 16%) for ICSI cycles with frozen/thawed spermatozoa obtained during the previous fresh TESE [57]. Likewise, a recent meta-analysis showed that fertilization and pregnancy rates were similar when using fresh vs. frozen sperm from testicles, even in men with poor spermatogenesis [138]. In contrast, higher miscarriage rates and lower live birth rates were observed in patients who underwent ICSI cycles with cryopreserved spermatozoa vs. fresh spermatozoa from microTESE [139]. Similarly, in men with Klinefelter’s syndrome, the use of fresh testicular sperm provided higher pregnancy rates than frozen sperm (60% vs. 25%) [140]. These differences may be due to male-related parameters, such as FSH concentration, testicular volumes, and degree of spermatogenesis defect, in addition to the number of testicular samplings, sperm preparation techniques, method of sperm selection, woman’s preparation protocol, and experience of the assisted reproductive technology (ART) center [141]. Additionally, the retrospective designs of most studies, relatively small numbers of participants, and scarcely provided confounding factors limit the ability to achieve a definitive conclusion about fresh vs. frozen/thawed TESE. Overall, recent technological and methodological advances in sperm cryopreservation with minimal damage have allowed this option to be used safely and effectively in many ART attempts.

### 3.2. Whole Tissue or Isolated Spermatozoa

Experimental studies comparing the cryopreservation of germ cells after isolation or within testicular tissue showed that the latter provides a higher rate of cell survival and the tissue structure remains unchanged [142,143]. As a common use, cryostorage of testicular tissue as fragments after gentle mincing was found to be more effective for maintaining viability after thawing than cell suspension [144]. However, in humans, there is no consensus regarding the storage of post-TESE germ cells as whole tissue or cell solution. Although spermatozoa frozen in seminal fluid are more resistant to the freezing process than washed sperm [145], the progressive motility of the samples, whether with or without seminal plasma, did not change in sperm bank donations after long-term cryostorage [128]. Besides, in cases of NOA where the sperm number is very limited within testicular specimens, it is preferable to freeze the whole tissue samples intact, as minimal processing of the tissue may allow the sperm to better maintain their surveillance and motility after thawing [146,147]. According to a comparative study, there were no significant differences between ICSI cycles with fresh or testicular spermatozoa cryopreserved in whole tissue in terms of clinical pregnancy rates (26% and 27%, respectively) and delivery or ongoing pregnancy rates (21% and 9%, respectively) [148]. However, clinical results similar to those with fresh sperm were also obtained in cases in which a few isolated testicular spermatozoa were frozen in glycerol-containing cryopreservation medium [149]. Cryopreservation of isolated testicular spermatozoa in straws is a widely used method all over the world [19,148,150,151]. Regarding such storage, it has been suggested that fully filling a small-volume straw would provide better protection than filling a larger straw up halfway, as the negative effect of the partial volume is associated with free radicals and intratubular pressure as well as biological effects [128].

Storing testicular tissue samples from which spermatozoa can be retrieved provides the opportunity not only for use in subsequent ICSI cycles but also to perform both tissue and cell therapy for in vitro maturation of immature germ cells. Thus, cryostored tissue samples can be used for future testicular grafting or organ culture procedures as well as for testicular transplantation or in vitro maturation with germ cells isolated from the tissue. At the same time, the niche microenvironment formed by Sertoli, Leydig, peritubular, and somatic cells in the preserved testicular tissue allows germ cells to maintain their viability and function after thawing [152,153]. On the other hand, cryopreservation of homogenized isolated spermatogenic cell fractions has been shown to preserve cell viability more than whole testicular extracts [154]. This can be explained by the detrimental role of toxins in the tissue suspension, which are secreted by non-germ cells. However, according to the available data, it may be more efficient to store a small number of low-quality spermatozoa while preserving tissue support. Conversely, for spermatozoa retrieved in sufficient numbers and with excellent motility, cryostorage may be preferred after isolation. Nevertheless, the choice between cryopreservation with isolated cells or using whole tissue should be made based upon personal experience and the facilities of the institution. However, in cases where extremely few spermatozoa have been dealt with, sufficient data has not yet been collected to make a definite decision on which method is more effective. The experience gained from the use of novel technological resources in laboratory procedures will be the deciding factor for this issue.

### 3.3. Cryopreservation Methods

Cell survival after cryopreservation depends on how minimally intracellular ice crystals are formed. By using cryoprotectants and adjusting the freezing/warming rate, it is possible to reduce the amount of intracellular water and, eventually, ice formation. Different cryopreservation methods have been developed with different freezing rates, cryoprotectant concentrations, and temperature reductions. Conventional freezing is a manual storage technique using liquid nitrogen and can be done using fast or slow freezing methods or with a programmable freezer [155]. Vitrification is an ultra-fast method developed as an alternative to rapid freezing in nitrogen vapor [156]. Unlike the gradual cooling in the conventional freezing method, during vitrification, the sample is submerged directly and quickly in liquid nitrogen at −196 °C without being exposed to its vapor. A comparative study demonstrated that progressive sperm motility and vitality were higher after conventional rapid freezing than after vitrification, whereas vitrification was more successful if normal morphology was the criterion [157]. Despite the widespread preference of the conventional technique in ART clinics, vitrification is recommended as a fast, practical, and low-cost method [158,159]. Rapid freezing also prevents damage due to intracellular ice crystallization during cooling [160]. According to a recent meta-analysis on cryopreservation of spermatozoa, vitrification is superior to conventional freezing in terms of total and progressive motility, but the post-thawing DNA fragmentation index and morphology are similar for both methods. Although the most commonly used method for cryopreservation of samples from TESE is conventional rapid freezing in liquid nitrogen vapor [161], vitrification has also been encouraged, especially in the freezing of rare spermatozoa. Due to the absence of permeable cryoprotectants and the sudden exposure to cold, vitrification has become advantageous in the storage of surgically retrieved spermatozoa concentrated in very small volumes [162]. Limited numbers of studies with NOA cases have reported healthy pregnancies in ICSI cycles using testicular spermatozoa frozen by vitrification [151,163,164]. For this purpose, specially designed tools have been developed that can allow freezing of a small number of spermatozoa [165]. Spis et al. compared the results of TESE spermatozoa cryopreserved by conventional freezing with those frozen using cryoprotectant and cryoprotectant-free vitrification methods [163]. They vitrified spermatozoa in 50-μL plastic capillaries in culture medium with 0.25 M sucrose. After a capillary was placed in a straw, it was plunged into liquid nitrogen and cooled at 600 °C/min. When thawed, the motility of small volumes of vitrified spermatozoa was found to be significantly higher than that of spermatozoa frozen using the conventional method (8.0% vs. 0.6%). The vitrification-in-straws method has also yielded highly efficient results in the cryopreservation of human testicular diploid germ cells in terms of recovery and viability [166]. However, the efficiency of vitrification varies depending on protocol and sperm quality. The roles of slow-programmable freezing [167], ultra-rapid freezing [168], solid-surface vitrification [169], and cryoprotectant-free freezing [170] on the molecular and structural effects of cooling have been further investigated in other studies.

### 3.4. Choosing the Proper Cryoprotectant

Another important factor that can determine sperm retrieval efficiency and quality in cryopreservation is the protectant used. In addition to permeable cryoprotectants such as glycerol, dimethyl sulfoxide (DMSO), dimethyl acetaldehyde, propylene glycol, ethylene glycol, and 1,2-propanediol, which are routinely used in cryopreservation, non-permeable agents—glucose, sucrose, egg yolk citrate, albumin, polyethylene glycol, and trehalose—have also been validated for use [171,172]. Pregnancy has been achieved for decades with frozen/thawed testicular spermatozoa using glycerol as a cryoprotectant [173,174]. Although experimental studies have shown the protective effect of glycerol on sperm structure to be better than that of dimethyl sulfoxide (DMSO) [175], Keros et al. recommended DMSO as an ideal penetrating agent if permeable protectants are to be used in cryopreservation of testicular specimens [176]. Additionally, compared to glycerol DMSO was found to be the most effective protectant in cryostorage of immature testicular samples [144]. In fact, immature testis tissue was demonstrated to be more susceptible to cryoprotectant toxicity with cell-specific sensitivity. However, when human testicular tissue was stored separately in DMSO, 1,2-propanediol, ethylene glycol, or glycerol using the slow freezing method, 52% to 58% of the cells remained alive, indicating that the selection of cryoprotectant did not affect viability after thawing [177]. However, the use of testicular samples with normal spermatogenesis and the lack of recovered cell numbers limit the interpretation of these results. Furthermore, different results have also been reported with regard to the cryoprotectant selection depending on the patient’s age, the agents being compared, and the procedure used [144].

Since permeable protectants easily pass through the cell membrane, they form an osmotic gradient that draws water out of the cell, thereby preventing the formation of intracellular ice crystals [162]. However, these reactions can have toxic effects on the sperm plasma membrane due to lipid peroxidation as well as the stress created during processing [178]. In contrast, non-permeable cryoprotectants cannot pass through the cell membrane due to their high molecular weight, and extracellular solute accumulation allows the intracellular water to be discharged; thus, dehydration occurs in the cell. Medrano et al. reported the first birth of a healthy infant following ICSI using the permeable cryoprotectant-free sperm vitrification protocol [179]. Furthermore, the use of non-penetrating cryoprotectants at low concentrations has also pioneered the design of minimal-sized carriers to reduce fluctuations in cell volume, thus initiating a new era in the storage of small volume sperm obtained from TESE material. Subsequently, Spis et al. reported better sperm quality in epididymis and TESE spermatozoa when they used the cryoprotectant-free vitrification method compared to the conventional method using permeable cryoprotectants, and ICSI with cryoprotectant-free vitrified spermatozoa resulted in delivery of healthy babies [151]. Similarly, sperm droplets frozen on cryoloops showed that the motility of vitrified spermatozoa in the absence of cryoprotectant was not different from that of conventional freezing with protectants and did not affect DNA integrity [180]. In current practice, low chemical toxicity and limited osmotic shock have made non-penetrating cryoprotectants a feasible option for testicular sperm storage. As an alternative, combining two different protectants can reduce the marked toxic effects of cryoprotectants [180,181]. For this purpose, loading a small number of spermatozoa on a cryoloop in a 50:50 mixture of test yolk buffer with glycerol and modified human tubal fluid medium supplemented with 6% Plasmanate was shown to preserve sperm viability and function [182]. Various protectant mixtures have been investigated to reduce the concentration of compounds in order to ensure their maximum efficiency without reaching toxic levels [165].

### 3.5. Cryopreservation of Very Few Spermatozoa

In some TESE cases, despite all efforts, only an extremely small number of sperms can be extracted. Such situations pose a serious problem—namely, the loss of the few sperms after freezing/thawing processes [41,135]. There is an ongoing effort to develop optimized freezing protocols and effective technologies that will shorten the post-thaw search time and minimize sperm loss. However, current technologies have given patients with NOA the opportunity to retain their fertility by implementing cryopreservation of even a single sperm [165]. Freezing in microstraws was proposed as an option for the cryostorage of few spermatozoa. After thawing, microstraw samples reached a significantly higher rate of sperm motility than the traditional straw samples [183]. As microstraws are thinner and a very small volume of medium is loaded, this method allows faster freezing. Likewise, Desai et al. isolated motile spermatozoa individually with an ICSI needle, loaded them into a capillary tube, and then carefully inserted the tube into the outer straw [184]. Samples were frozen using the conventional method by plunging into liquid nitrogen. The post-thaw recovery rate ranged from 33% to 100%, and pregnancy was successful. In experimental studies, sperm vitrification in straws was demonstrated to preserve motility better than the spheres method where permeable cryoprotectants were not used [185].

In extreme cases, storage in empty zona, non-biological carriers, or vitrification devices has also been suggested for freezing and storing lone or very few sperm cells. A novel option for preserving only a few spermatozoa is to reduce the volume of cryoprotectant-added media and ensure efficient identification after thawing. For this purpose, the insertion of spermatozoa into encapsulated porous capsules has been suggested to allow easy visualization and manipulation during cryopreservation processes. It was shown that oocyte zona pellucida (ZP) can be used as a vehicle following removal of cellular material. Subsequently, empty ZPs prepared from humans and mice were used as frozen vectors in experimental studies [165,186,187]. Walmsley et al. reported the first live human birth associated with this procedure using testicular sperm [188]. Although an animal ZP is a biological carrier, it has not been widely used due to its low availability and bioethical issues as well as procedural problems (e.g., the risks of residual host DNA fragments and foreign DNA transfer and impaired sperm quality due to an artificially induced acrosome reaction) [186]. As a solution to the constraints of human oocyte use, algae Volvox globator spheres offered a promising approach to the cryopreservation of a single motile sperm, but the possibility of foreign DNA transfer was questionable [189]. With the encouragement of these studies, fabricated non-biological carriers, such as alginic acid capsules [190], agarose capsules [191], and hyaluronan-phenolic hydroxyl microcapsules [192], have also been developed as empty capsules for single-sperm freezing. However, empty capsules are not widely used due to the complexity of their fabrication and problems associated with their storage.

Although non-biological spheres give hope for future use due to their low toxicity, lack of ethical problems, and acceptable post-thawing motile sperm retrieval and survival results, the lack of existing clinical outcomes makes it difficult to reach a definitive conclusion. Recently, non-labor, non-biological, bioethically acceptable, and inexpensive commercialized alternative devices have been designed for the cryopreservation of a small number of spermatozoa. The Cryotop device developed for this purpose is formed by inserting a fine polypropylene strip on which sperm droplets are placed into a cover straw [193]. Recently, Ohno et al. evaluated the efficiency of cryopreservation of three or fewer spermatozoa loaded on the Cryotop using a modified permeable cryoprotectant-free vitrification method [194]. Clinical pregnancies resulting from vitrified spermatozoa from the ejaculate, fresh spermatozoa from the ejaculate, and vitrified spermatozoa from the testis were found at similar rates (25%, 24%, and 16%, respectively). Only the sperm survival rate and the oocyte fertilization rate were found to be significantly lower in attempts using vitrified spermatozoa from the testis compared with vitrified spermatozoa from the ejaculate. The researchers concluded that this technique was particularly effective for the cryopreservation of samples with fewer than 10 testicular spermatozoa. Cryoloops represent another efficient and non-labor-intensive method for the cryopreservation of individual spermatozoa [195]. With the aid of micromanipulator equipment, selected spermatozoa loaded onto an open cryoloop are enclosed in a vial and stored in liquid nitrogen. After loading 5–10 spermatozoa into each loop, total recovery and post-thaw survival rates were reported as 68% and 70%, respectively [182]. Individual testicular spermatozoa cryopreserved on cryoloops have also been shown to successfully fertilize oocytes [196]. Although open systems allow rapid cooling, direct exposure of semen to liquid nitrogen poses a potential risk of contamination [159]. Another closed system developed for sperm cryopreservation is the Cell Sleeper. Initially, the recovery and viability rates of individually vitrified spermatozoa using the Cell Sleeper were reported as 100% and 72%, respectively [194]. In this method, individual spermatozoa are transferred into freezing medium on an inner tray with a micromanipulator needle. Once the tray is placed in a vial, the cap is screwed on and it is immersed in liquid nitrogen. In a case of NOA, the authors reported that when ICSI was performed with spermatozoa frozen using the Cell Sleeper, five out of six oocytes were fertilized, resulting in the birth of a healthy baby [193]. Likewise, various carrier devices based on the same principle, such as Cryolock [197], Cryoleaf [198], Cryopiece [199], and SpermVD [163], have also been designed and applied in vitrification.

During the thawing of the vitrified samples, the warm-up speed should be high so that the frozen water inside spermatozoa can switch to the liquid phase without crystallization. The optimal thawing temperature to maintain membrane integrity and sperm function is still the subject of investigation. Depending on the methodological differences used in vitrification, temperatures ranging from 37 °C to 44 °C have been attempted [157,200,201]. However, there are limited cohort studies evaluating the sperm retrieval, fertilization, and pregnancy rates of these methods in IVF following cryopreservation. Moreover, the need for skilled personnel in handling the devices and the prolonged time of the laboratory processes have restricted the routine use of manufactured carriers. Although the fact that these novel devices can store a single spermatozoon has met an important need with respect to the management of patients with NOA, further research confirming their feasibility and efficiency is needed to adapt these systems for clinical practice.

### 3.6. Drawbacks of Sperm Cryopreservation

To some extent, cooling, thawing, and exposure to cryoprotectants may cause destructive changes to sperm function and structure [135]. Most importantly, in cases of NOA, post-thawing viability may be reduced significantly, since sperms obtained by TESE have reduced resistance to mechanical, thermal, and osmotic stress due to structural or functional defects [202]. For this reason, if very few spermatozoa were found in the initial search, the possibility of their disappearance after thawing (and, consequently, cancellation of the ICSI cycle) may be encountered. Up to a two-fold increase in the number of immotile sperm was found in frozen samples [137,203]. Sperm motility has also been shown to decrease from 50% in fresh semen to 7% in frozen/thawed samples [46]. Additionally, the broken neck abnormality observed after thawing is related to damaged centriole structure due to cryoinjury, causing fertilization failure in ICSI [19]. Quality impairment of frozen/thawed sperm is mostly attributed to DNA damage, which determines embryo quality and viability [204]. In fact, in cases of TESE, decreased concentration and reduced mitochondrial activity, as well as a significant increase in DNA fragmentation and reactive oxygen species (ROS) production, were reported in post-thawed sperms in comparison to freshly recovered samples [155]. Generation of ROS during sperm cryopreservation may damage many cellular components (e.g., membrane, cytoskeleton, DNA, and mitochondria), resulting in loss of function and genomic instability [205]. Thus, the decrease in sperm competence caused by excessive intracellular oxidative stress is accompanied by impaired fertilization and poor embryo development [206]. Although it is widely accepted that the freezing process causes remarkable changes in sperm parameters, the effects of various techniques may differ [207,208]. For example, in comparison to slow programmable freezing, rapid freezing has been found to be more advantageous in terms of post-thawing motility and cryosurvival [209]. With this knowledge, the advantages and disadvantages of each cryopreservation method should be evaluated on an individual basis.

When evaluating the drawbacks of cryopreservation, one should also take into account the functional capacity of sperm retrieved from the testicles, which are the products of defective spermatogenesis. Freezing of low-quality testicular specimens containing non-motile spermatozoa or spermatozoa with poor morphology has demonstrated a negative impact on embryo quality [210]. Moreover, Wu et al. showed that many sperms obtained from the testicles remained in the immature stage and the spermatozoa carried cytoplasmic droplets in the neck and mid-piece [41]. In fact, when compared with ejaculated sperm, miscarriage rates were found to be higher in cases of ICSI using testicular sperm. Chromosomal aneuploidy is more likely to be encountered in testicular sperm from patients with NOA [211,212]. Moreover, cryopreservation of testicular cells or tissues poses the risk of the presence of residual cancer cells during the transplantation of autologous cells in patients scheduled for oncotherapy, which may trigger the disease [153]. However, the absence of such a risk, at least in experimental studies, has been demonstrated by the lack of increase in cancer incidence or survival reduction after testicular transplantation of propagated spermatogonial stem cells (SSCs) [213]. Furthermore, the contamination risk of frozen sperm samples has not yet been completely resolved. The source of contamination may be the sample itself or the infected frozen carrier, liquid nitrogen, or storage tank. As single-sperm cryopreservation is performed with a more controlled technique, particularly in closed systems, a potentially reduced risk of contamination is expected [165].

Notably, in recent decades, some environmental, occupational, and lifestyle-related risk factors have accompanied the declining trend in semen quality through certain genetic and metabolic pathways whose exact causes have not been fully elucidated [214]. Therefore, when interpreting IVF results of fresh vs. frozen sperm, the quality of the sperm chosen for injection into the oocyte and the presence of DNA damage should be considered as the deciding factors. These parameters may also have potential impacts on the consistency of the results. Indeed, unlike in the aforementioned studies, Semião-Francisco et al. reported that the pregnancy and miscarriage rates of sperm taken from the testicles, whether in obstructive or NOA cases, did not differ significantly [215]. Likewise, pregnancy rates remained similar whether frozen or fresh TESE samples were used (32.1% and 35.7%, respectively; *p* = 0.62) and did not show a significant correlation with the use of motile or immotile sperm (46.3% and 66.7%, respectively; *p* = 0.59) [216]. Regarding the inconsistencies between results, a methodological analysis evaluating the success of TESE results in men with NOA emphasized the current lack of knowledge, especially regarding the quantity and quality of sperm retrieved [217]. Recently, OMICS technologies such as genomics, transcriptomics, proteomics, and metabolomics, in combination with advanced bioinformatics technology (e.g., Illumina RNA sequencing, high-throughput next-generation sequencing, multiplexed enzyme-linked immunosorbent assays), have provided an extensive opportunity for research on how cryopreservation affects sperm structure and function [218]. Nevertheless, no matter how few sperm can be retrieved, it has been suggested that cryopreservation should be considered as a feasible method in every surgical sperm retrieval case [148]. Beyond all, carefully and accurately recording data and respecting ethical considerations and legal regulations are also essential steps to maintaining trust.

### 3.7. Minimizing the Harmful Effects of Cryopreservation

When it comes to dealing with cellular damage due to cryopreservation, recent nanotechnological trends are promising [219]. Due to their unique physical and chemical nature as well as their low toxicity, nanoparticles can increase the absorption and bioavailability of protective ingredients by spermatozoa. Supplementation of the tris-based SHOTORTM extender with zinc and selenium nanoparticles was shown to enhance sperm progressive motility, vitality, and membrane integrity after cryopreservation by reducing apoptosis and lipid peroxidation [220]. Similarly, in testes, the use of nanoparticles containing necrosis-inhibitory factors greatly improved tissue integrity and survival of germ cells [221]. Artificial or natural nanovesicles, such as liposomes and exosomes, are also promising measures for protecting sperm from the harmful consequences of freezing and thawing [222]. After exosomes are secreted from the cell, they are taken in by the target cells and transfer their protein and RNA contents to those cells. In experimental studies, seminal plasma and mesenchymal stem cell-derived exosomes have been shown to improve the quality of frozen/thawed spermatozoa [223,224]. A liposome is an artificially manufactured, exosome-like, spherical nanovesicle with a lipid bilayer that can transfer cryoprotectant contents to a spermatozoon by fusing with the sperm plasma membrane [225]. In animal studies, liposomes were shown to increase sperm motility and viability, strengthen the membrane structure, and improve fertility [226]. Likewise, testicular experimental studies involving gene technology have shown that knockout serum replacement (KSR) supplement, a chemically defined medium, provides a cryoprotective effect comparable to that of conventional sera but more consistent in quality [227]. The beneficial effects of using antioxidants in seminal plasma (but not yet in testicular tissue) on sperm parameters and ROS production during the freeze/thaw process were widely discussed in a systematic review and meta-analysis by Bahmyari et al. [228]. The addition of exogenous antioxidants into the freezing medium may ameliorate sperm damage by reducing the oxidative stress caused by cryopreservation. For frozen semen, resveratrol, lycopene, vitamin E, and quercetin are the most commonly used agents to protect sperm from ROS damage [229,230]. However, further studies are needed to evaluate how nanoparticles and additives affect clinical practice outcomes and embryo development when used for small numbers of poor-quality testicular spermatozoa.

## 4. Processing Immature Germ Cells

Despite extensive searching, mature spermatozoa can be obtained with TESE in only approximately 40% to 60% of cases of NOA for use in ICSI [231,232]. In the remaining cases, round spermatids were attempted as a last resort, although until recently, the results were not satisfactory enough to encourage routine practice [233]. However, in 2015, Tanaka et al. reported 14 healthy babies born with round spermatid injection (ROSI) in oocytes previously activated by electric current; 3 years later, from the 2-year follow-up results of 90 ROSI babies, it was determined that round spermatids enabled patients with NOA to have their own genetic offspring [23,234]. Subsequently, Papuccu et al. reported their results of 472 couples who underwent 904 cycles using elongating (Sb2) spermatids for the ROSI technique and achieved a 9.6% ongoing pregnancy rate [235]. Since transformation of immature germ cells into spermatozoa with fully developed flagella has had limited success in in vitro experiments, culturing samples to achieve at least haploid round spermatids may have wider clinical application [236].

If testicular tissue samples from TESE do not contain spermatozoa, various approaches have been described for in vitro maturation of early stage germ cells. For in vitro maturation, either (1) testicular tissue is used in whole pieces while preserving its three-dimensional (3D) structure or (2) after isolation and purification, different cell types are exposed to culture conditions in which the spermatogenic process is recreated. In TESE-negative cases, it makes sense to try culturing small fragments of testicular tissue or intact pieces of tubules first. However, since the limited diffusion rates of the tissue do not allow tissue viability to be maintained over a long period, it is challenging to culture the tissue as a whole. After long-standing efforts, in 2011, Sato et al. reported healthy offspring from haploid cells developed from testicular fragments cultured on agarose gel in modified Minimum Essential Medium (α-MEM) supplemented with knockout serum replacement [237]. Others have also shown culturing of frozen/thawed testicular tissue on agarose gel to restore spermatogenesis up to haploid spermatids, leading to offspring [238]. Furthermore, from seminiferous tubule segments cultured in chitosan hydrogel bioreactors, the development of spermatids and spermatozoa were achieved on days 34 and 55, respectively [239]. In another organotypic culture system described for human immature testicular tissue, germ cells were shown to differentiate up to round spermatids within 16 days [240]. However, the inability of organ culture systems to restore spermatogenesis in cryopreserved human testicular specimens has also been reported [241]. These contradictions in results may be due to the fact that culture methods are not yet fully optimized or that the nature of subcellular defects is different [242]. Later, developed microfluidic technology further improved organotypic culture systems, allowing ex vivo sustainability of the structure and viability of germ cells in testicular tissue for producing mature sperm [243]. As an option for in vitro maturation of isolated germ cells, 2D culture systems have been developed to support enzymatically digested testicular cell suspensions. In 2D cultures, isolated SSCs are maintained either on feeder cells or on mixed cell populations co-cultured with somatic cells [244]. Different feeders, including SIM mouse embryo-derived thioguanine and ouabain resistant (STO), mouse embryonic fibroblast, bovine Sertoli cells and laminin-coated plate were used to support spermatogenesis [245]. Apart from feeders, different culture systems, such as human amnion mesenchymal stem cells [246], in vitro reprogramming of fibroblasts to human induced Sertoli-like cells [247], and isolated cell culture with growth factor supplementation [248], have been defined to support in vitro spermatogenesis. Nevertheless, 2D culture systems provided in vitro restoration of spermatogenesis and supported the development of haploid spermatids with fertilization potential [249]. Experiences gained from previous studies ultimately led to the development of artificially constructed 3D structures [250]. By creating structures that mimic the composition of the main testicular components, 3D cultures allow immature germ cells to be reconstructed similarly to their original tissue architecture, thereby allowing further maturation [251,252,253,254]. Although this system has the ability to direct the differentiation of germ cells, microenvironmental conditions favorable for complete maturation must also be achieved.

In addition to attempting complex and intricate methods for in vitro maturation of immature germ cells from testicular tissue, there is a need for simple methods that can be used more easily in clinical practice. When Aslam et al. compared suspensions of mixed cell populations and isolated homogeneous populations of spermatogenic cells prepared from testicular tissue, they showed that most of the isolated round spermatids developed tails and remained intact and viable for 72 h in modified Eagle’s minimum essential medium with no hormonal supplementation [154]. However, since they used a mixture of obstructive and non-obstructive tissue samples, the contribution of in vitro culturing to the development of flagella in immature germ cells in cases of NOA is not clear. Similarly, it has been shown that human round spermatids can mature up to spermatozoa when cocultured on Vero cell monolayers [255]. Other researchers have also verified the maturation of primary spermatocytes into haploid spermatids through in vitro coculture with Vero cells [256]. Subsequently, round spermatids generated from human SSCs were shown to fertilize mouse oocytes [248]. However, even without a co-culture, in vitro hormonal supplementation has been demonstrated to be capable of providing sufficient support to mature premeiotic germ cells [257]. Thus, culturing testicular samples from patients with NOA in medium containing recombinant FSH and testosterone for 48 h transformed FISH-proven primary spermatocytes into mature round spermatids, after which injection into the oocyte resulted in healthy offspring [258]. It has been shown that hormones added to in vitro culture medium in cases of NOA not only accelerate spermiogenesis but also improve apoptosis-related cell damage in enhancing the reproductive performance of germ cells [259]. Contrary to most studies indicating that FSH and testosterone added to testicular culture media play a role in the development of different stages of in vitro spermatogenesis, it has also been suggested that their supplementation does not induce meiotic and post-meiotic cells and therefore cannot differentiate premeiotic germ cells [260]. Differences in germ cell development in in vitro maturation studies may be due to insufficient support of established culture conditions. The maintenance of healthy spermatogenesis from SSCs can only be achieved with the support of a complicated and precise “niche” microenvironment [261]. Sertoli cells establish the most important component of the “niche,” and by producing growth factors and cytokines, they regulate proper self-renewing and differentiation of SSCs, transition to meiosis, and, finally, differentiation of round spermatids into spermatozoa [262].

Under proper culture conditions, reaggregation of Sertoli cells forms organized monolayer structures. Therefore, in most of the in vitro maturation studies performed on testicular tissue samples, a co-culture with Sertoli cells has been used effectively to provide structural and nutritional support for differentiation of germ cells [263,264,265]. In the co-culture of round spermatids and Sertoli cells, it has been shown that supplementation with recombinant FSH and testosterone contributes significantly to the differentiation of round spermatids into elongating spermatids [266]. However, other studies have also reported that no matter how much FSH stimulation in organotypic cultures of immature testicular tissue increases the percentage of premeiotic cells, it does not allow for further maturation [242]. Some similar studies have also confirmed that FSH supplementation in cultures does not support post-meiotic maturation [267]. Actually, the underlying mechanism for the contradictions in the reports may be the impaired ability of testes to respond to the endogenous hormonal milieu due to compromised androgen receptor and FSH receptor (FSHR) signaling pathways [268]. In fact, when compared to obstructive azoospermia, the FSHR expression level in isolated and purified Sertoli cell cultures was found to be 2.7 times lower in the NOA group; hence, it has been claimed that there may be an altered Sertoli cell response to in vitro FSH stimulation [269]. Alternatively, without the need for hormone supplementation, spermatogenesis from testicular SSCs to fertility-competent sperm formation could be induced in organ cultures using different techniques [270,271]. Considering this fact, before choosing a method to be used in co-culture studies with Sertoli cells, it is important to investigate the hormone/receptor interaction along with the response to FSH and testosterone.

To date, a number of studies have been conducted on the development of many different culture systems, with varying levels of success. Furthermore, with their innovations in in vitro germ cell maturation, advanced technology products created in the field of regenerative medicine using cell/tissue culture, biomaterials, and bioactive products have become promising treatment alternatives for patients with NOA [272,273]. However, before suggesting potential clinical uses of haploid male gametes exposed to in vitro manipulations, further analyses of fecundity, epigenetic consequences, and safety are essential.

## 5. Conclusions

The primary outcome associated with the efficiency of ARTs is successful, healthy live births. In addition to allowing only a small amount of sperm retrieval, cases of NOA require a more demanding process in the treatment of infertility due to the fact that the available sperm are also products of impaired spermatogenesis. The whole process begins with collection of the highest-quality surgical specimens possible. Following microTESE, with its verified efficacy, researchers have attempted sophisticated techniques such as Raman spectroscopy, multiphoton microscopy, and full field optical coherence tomography to identify testicular tubules with spermatogenesis. As further developments, laser-assisted sperm selection and microfluidic systems appear to be promising for extracting viable spermatozoa from surgically removed testicular samples. Moreover, there is an ongoing effort to develop optimized freezing protocols and effective technologies that will allow patients with NOA to retain their fertility by implementing cryopreservation of even a single sperm. However, the initial promising results of all of these developments must be confirmed by large studies in the context of clinical practice. The use of nanoparticles for in vitro maturation of germ cells is also another promising innovation, as it will allow previously unsuccessful patients with NOA to have children using their own biological material. Undoubtedly, the clinical consequences of all of these manipulations that could result from potential changes in the offspring’s genomes must be followed very carefully.

## Figures and Tables

**Table 1:** Clinical results of enzymatically tissue processing methods for sperm recovery by TESE.

Compound	Control	Results	Refs.
DNase plus collagenase type IV	Mechanical searching	Increased SRR 9% of cases where no spermatozoa were found after mechanical searching.	[33]
DNase plus collagenase type IV	Mechanical searching	Increased SRR 26% of cases where no spermatozoa were found after mechanical searching	[34]
DNase plus collagenase type IV	Mechanical searching	Increased SRR 33% of cases where no spermatozoa were found after mechanical searching	[35]
DNase plus collagenase type IV	Mechanical searching	Increased SRR 7% of cases where no spermatozoa were found after mechanical searching	[36]
Collagenase type IV and collagenase type IA	Untreated samples	Vitality in control, collagenase IV and IA; 74.7%, 84.9% and 79.5%, respectively, motility; 86%, 86% and 71%, respectively (*p* > 0.05). Recovered spermatozoa by collagenase IV and IA; 0.34 × 10^6^ and 0.22 × 10^6^, respectively *p* = 0.017	[28]

**Table 2:** Clinical results of motility enhancers for sperm recovery by TESE.

Compound	Control	Results	Refs.
Pentoxifylline	Hypoosmotic test	Increased fertilization rate (62% vs. 41% *p* < 0.05) Increased pregnancy rate (32% vs. 16% *p* < 0.05)	[53]
Pentoxifylline	Untreated immotile sperm	Initiated motility in 95.7% of the samples. Increased fertilization rate (66% vs. 50.9%; *p* < 0.005) Increased mean number of embryos per cycle (4.7 vs. 2.7 *p* < 0.01).	[55]
Pentoxifylline	In-group	Induced additional motility in 33.3% and 69.3% of cases where fresh and frozen samples were used, respectively.	[57]
Pentoxifylline	Untreated immotile sperm	Initiated motility in 70.8% of the samples.	[42]
Theophylline	Untreated motile or immotile sperm	Improved motility in 98.5% of the cases. Increased fertilization (79.9% vs. 63.3% *p* < 0.001) and pregnancy (53.9% vs. 23.8% *p* < 0.05) rates.	[45]

**Table 3:** Clinical results of short-term testicular tissue culturing.

Time of in Vitro Culture	Control	Results	Refs.
24 h	0 day	Improved motility from 13% to 76% (at 25 °C) and 67% (at 37 °C) (*p* = 0.01)	[72]
24 h supp recFSH	24 h simple medium	recFSH supplementation improved motility to 70.4% vs. 32.9%, fertilization 68.8% vs. 42.1%, implantation per embryo 20.1% vs. 13.2%, and clinical pregnancy 47.9% vs. 30% (*p* < 0.005)	[73]
24 h—72 h	0 day	After 24 h in culture, a marked increase of 5–8% in motile sperm was observed and a maximum motility rate appeared between 48 and 72 h of culture (*p* < 0.05)	[41]

**Table 4:** Clinical outcomes of different methods for viable sperm recovery by TESE. *NS: no significant.*

Method	Comparison	Results	Refs.
HOST	Sperm morphology	Significantly higher fertilization 43.6% vs. 28.2%, pregnancy 27.3% vs. 5.7% and ongoing pregnancy 20.5% vs. 2.9%.	[79]
	Testicular vs. ejaculated spermatozoa	Fertilization 30.1% vs. 42.7% (NS), pregnancy 16.7% vs. 13.3% (NS) and delivery/ongoing pregnancy 8.3% vs. 6.7% (NS), respectively.	[80]
Sperm tail flexibility test	Motile vs. immotile sperm selected by the test	In frozen-thawed samples; fertilization 74.3% vs. 65.7%, and pregnancies three vs. two, respectively (NS). In fresh samples; fertilization 64.4% vs. 73.4%, and pregnancies nine vs. three, respectively (NS).	[94]
Laser-assisted sperm selection	Random sperm selection	Higher fertilization 45.4% vs. 20.4% *p* < 0.0001, cleavage 64.4% vs. 30.6% *p* < 0.0001, and take-home-baby rate 9.0% vs. 5.9%.	[103]
Sperm birefringence	Normal motility/morphology	Improved grade I/II embryo 71.2% vs. 63.4% and pregnancy 46.6% vs. 33.3%, respectively.	[107]
	Routine sperm selection	Improved pregnancy 58% vs. 18% *p* = 0.053, implantation 42.1% vs. 12.5% *p* = 0.049 and ongoing pregnancy 58% vs. 9% *p* = 0.018, respectively.	[108]
Microfluidics-assisted sperm sorting	Standard sample processing	Improved sperm yield 13.5 sperm per min vs. 1.52 sperm per min.	[113]
Fluorescence-activated cell sorting	Standard sample processing	Improved sperm recovery 50% vs. 38%, respectively.	[121]

## Data Availability

The data presented in this study are openly available in PubMed, PubMed Central (PMC) https://pubmed.ncbi.nlm.nih.gov/.
